# Distinct patterns of emotional and behavioral change in child psychiatry outpatients during the COVID-19 pandemic

**DOI:** 10.1186/s13034-022-00441-6

**Published:** 2022-02-17

**Authors:** Alysa E. Doyle, Mary K. Colvin, Clara S. Beery, Maya R. Koven, Pieter J. Vuijk, Ellen B. Braaten

**Affiliations:** 1 Department of Psychiatry, Massachusetts General Hospital, Boston, MA USA; 2 Harvard Medical School, Boston, MA USA; 3 Center for Genomic Medicine, Massachusetts General Hospital, 185 Cambridge St CPZN 6.240, Boston, MA 02114 USA

**Keywords:** COVID-19 pandemic, Child and adolescent psychiatry, United States

## Abstract

**Background:**

Studies are documenting the impact of the COVID-19 pandemic on youth mental health. We extended this literature by characterizing a child psychiatric outpatient sample in the United States during the middle of the 2020–2021 school year. We also used a computational strategy to identify distinct patterns of psychopathology symptom change and examined correlates and predictors of such change. Among potential predictors were cognition and clinical diagnoses, which have not been studied in this context previously.

**Methods:**

Participants were 171 youth (aged 10.6 ± 3.1) referred for neuropsychiatric evaluation who enrolled in research and whose parents filled out a survey on COVID-19. The questionnaire included eight psychiatric and six psychosocial domains rated retrospectively prior to the pandemic and currently at the time of evaluation. We examined change in severity of individual domains with Wilcoxon signed-rank tests. We used a latent profile analysis (LPA) to identify groups with distinct symptom change profiles. Using multinomial logistic regression, we examined potential predictors and correlates of LPA-derived groups. Models controlled for age, sex, and assessment date and corrected for multiple testing.

**Results:**

Although the majority of individual psychopathology domains were worse on average during the 2020–2021 school year, youth showed distincive patterns of symptom change. In addition to a large group (72.2%) with relatively stable symptoms and a small group (6.4%) that improved on most symptoms, there were two groups with different constellations of worsening symptoms. These latter groups both showed increased sadness, anxiety and oppositionality; however, one had increased hyperactivity/impulsivity and no change in hopelessness while the other showed greater hopelessness and no change in hyperactivity. Symptoms related to the distinguishable domains of these groups predicted group membership, and changes in screen time, conflict with parents and social isolation were correlates of worsening. Cognition and lifetime clinical diagnoses failed to predict group membership.

**Conclusions:**

In youth outpatients, psychiatric and psychosocial difficulties were worse on average during the school year following the spring 2020 COVID-19 lockdown; yet, some youth experienced greater and distinctive symptom change. A personalized approach to support may be needed as youth emerge from this period.

**Supplementary Information:**

The online version contains supplementary material available at 10.1186/s13034-022-00441-6.

## Background

The potential impact of the COVID-19 pandemic on youth mental health was anticipated early in 2020 [1] as communities around the world began to “lock down.” In the United States and other countries, schools were closed, recreational and extracurricular activities suspended, and even casual interactions were undermined by social distancing measures. Along with the disruption in learning and daily routines, the hardship of isolation was compounded by uncertainty, fear, and an economic and socio-emotional toll on the caregivers, teachers, and community organizations that often provide a buffer to children’s distress. The fact that youth, though less vulnerable in terms of their physical health, were at high risk with regard to their emotional and behavioral health was widely acknowledged [1, 2].

In time, studies from around the globe have begun to document an impact of these pandemic-related disruptions on the mental health of children and adolescents. Consistent with general trends in the empirical literature, there have been fewer published studies of youth compared to adults [3]. Nonetheless, emerging data in this age group have related the onset of the pandemic to increases in anxiety and depression [4, 5] behavioral difficulties [6], suicidality [7, 8], and psychiatric emergency room visits [9].

While such findings underscore the negative impact of the spring 2020 phase of pandemic on youth, data suggest that the emotional and behavioral sequalae of this period were not uniformly experienced. In spring 2020, for example, a survey of Canadian parents that addressed six common psychiatric dimensions [10] found that 70% of school aged youth had experienced worsening of at least one domain; yet, approximately half of the responses for each domain indicated no change or improvement in functioning. Such results highlight the need to better understand the variability of responses to the pandemic.

Already, data from the spring and summer of 2020 suggest that children and adolescents with pre-existing neuropsychiatric concerns may be vulnerable to difficulties [1, 10–14]. In a review of studies, Panchal and colleagues [15] concluded that youth with prior mental health concerns may be at increased risk for anxiety. Additionally, based on parent reports in a United Kingdom survey [13], youth with prior diagnoses of attention-deficit/hyperactivity disorder (ADHD) and autism spectrum disorder (ASD) both experienced greater pandemic-related emotional difficulties as well as greater “inattention/ hyperactivity” than youth without these diagnoses. Moreover, youth with diagnoses of ADHD were more likely to experience conduct problems, whereas youth with autism were more likely to have a decline in prosocial behavior. Thus, some domains of decline may relate to prior symptoms.

Yet, even among youth with mental health concerns, the emotional and behavioral response to the pandemic is not uniform. Indeed, a prospective survey of youth within a U.S. charter school found that mental health concerns measured prior to the pandemic predicted improved functioning during the late spring of 2020, presumably due to reduced academic and social stress when away from school. Given the data above in clinical populations, heterogeneity of response is likely, but not well understood. For example, in Cost et al.'s [10] Canadian survey, parent reported prior psychiatric diagnoses predicted improvement as well decline on different traits such as depression and irritability.

Gaining a better understanding of the variability in emotional and behavioral reaction to the pandemic in youth clinical samples is critical to mobilizing appropriate supports for youth who may be at greatest risk for difficulties. The current study aimed to advance the literature on clinical populations in several ways. First, we investigated the mental health impact of the pandemic on a generalizable outpatient child psychiatry sample in the United States, which to our knowledge has not been represented in prior studies. Second, we characterized the functioning of youth during the middle of the school year following the spring 2020 lockdown, thereby representing a later time period than prior studies of clinical populations. Third, we leveraged a computational strategy that allowed us to combine measurement of a wide range of psychiatric symptom domains with a child-centered approach to provide a snapshot of global functioning. Specifically, we used latent profile analysis (LPA) of changes in parent-reported retrospective pre-pandemic and current symptoms to identify groups of youth with similar patterns of multivariate change. Finally, we looked at predictors of patterns of change (i.e. predictors of LPA groups), including some that had not been used in prior studies, such as clinician-rated neuropsychiatric diagnoses and cognitive variables. Together, these analyses aim to extend our understanding of the variability of the mental health burden of the pandemic on clinical samples and potential predictors and correlates of different profiles of change.

## Methods

### Subjects

Participants were youth ages 6 to 17 who were consecutively referred for neuropsychiatric evaluation, enrolled in our larger, source study (the Longitudinal Study of Genetic Influences on Cognition; LOGIC), and agreed to complete an additional questionnaire about emotional and behavioral sequelae of the COVID-19 pandemic. Data collection for these analyses occurred during a six month period during the school year following the COVID lockdown (November 5, 2020 through May 4, 2021). Recruitment for the larger study occurs through the Learning and Emotional Assessment Program, a pediatric assessment clinic within the Psychiatry Department at Massachusetts General Hospital (MGH). Prior studies based on this growing cohort [16–18] have shown consistent rates of major diagnostic categories in these consecutively recruited outpatients, with approximately: 60% of youth meeting criteria for ADHD, 30–40% with anxiety disorders, 20% mood disorders, 11% ASD, and 3–4% psychosis.

Participation requires access to clinical data. Subjects are also asked for additional measures to create a uniform phenotype battery as well as a saliva sample for genomic analysis. DNA was not used for the current analyses. The Mass General Brigham Institutional Review Board approved the study procedures, which include parent written permission (youth 6–17) as well as child and youth written assent (ages 7–13 and 14–17, respectively).

## Measures

### Reaction to COVID-19 Questionnaire

We assessed youth reaction to COVID-19 through a parent questionnaire developed by our clinical research team. These data were collected using Research Electronic Data Capture (REDCap) tools hosted at Massachusetts General Hospital [19]. Questionnaires were sent via parent emails after enrollment in the LOGIC study. Questions relevant to the current analyses are provided in Additional file 1: Appendix S1. Questionnaires were collected between November 5, 2020 and May 4, 2021.

Our questionnaire was developed to assess 1) major psychiatric symptom domains as well as behaviors relevant to the psychosocial environment with potential to be impacted by the pandemic; 2) stressors and circumstances relevant to the pandemic with potential to impact children’s well-being. Individual psychiatric symptom domains were selected to represent domains of common internalizing and externalizing conditions that are typically assessed in youth omnibus measures. Behaviors relevant to the psychosocial environment and external stressors were selected based on clinical judgement as well as the emerging literature regarding risks to mental health during the pandemic [3, 20–22]. Questions have face validity for these purposes and show overlap with other questionnaires developed to assess the impact of the pandemic that have since been published. For example, five of the six dimensions of psychopathology assessed by the large Cost et al. [10] Canadian study were among the eight domains we examined. Additionally, the stressors we examined, including financial hardship (e.g. job/ income loss, food insecurity) social isolation and academic disruption have been assessed by other studies [2, 6, 23–25] including the large American ABCD cohort [26].

For the eight psychiatric symptom domains and six psychosocial domains, parents were asked whether these constructs were ever an issue for their child and if so were asked to rate them “during the school year prior to the pandemic” and “currently”, with options “not an issue”, “mild”, “moderate”, and “severe.” Psychiatric domains included: sad/depressed, worried/anxious, despair/ hopelessness, inattentive/ easily distracted, hyperactivity/impulsivity, irritable/ gloomy, and lacking interest in social interactions. Psychosocial domains included: arguing/conflict with friends, arguing /conflict with parents, wanting interactions but feeling isolated, spending too much time on electronic devices, using marijuana/ alcohol or nicotine products, engaging in risky behavior.

For change scores used in analyses, each level of change was represented as a point (e.g. pre-pandemic mild and current moderate = 1 point, pre-pandemic mild and current severe = 2 points). Additional background information was collected in the following domains: mental health services utilization, COVID-19 affecting loved one, financial security, child activities/ exercise, child isolation, schooling experience (including special education services), and parent–child relationship.

### Clinical diagnoses

Diagnostic procedures for the current sample were consistent with those in our larger source study and have been published previously [16, 17]. Lifetime DSM-5 diagnoses were made by licensed clinical psychologists who are MGH/ Harvard Medical School (HMS) faculty or pre- or post-doctoral clinical psychology fellows under their supervision. As our source clinic is an accredited academic training site, accurate and thorough diagnosis is a key component of youth evaluations. Diagnoses are made based on clinician’s judgement that full DSM-5 criteria are met based on the following sources of information: clinical interviews with a parent/ legal guardian and the patient, review of available medical and school records, and results of omnibus and targeted behavioral rating scales (including the Child Behavior Checklist/6–18 or Adult Behavior Scale, and the Child and Adolescent Symptom Inventory-DSM 5 edition).

Only lifetime diagnoses of ADHD and ASD were used in the the current analyses due to their requirements for longstanding patterns of behavior such that they could definitively be tied to the pre-pandemic period. In our source study, diagnoses of ADHD and ASD were previously shown to have high inter-rater reliability (Cohen’s kappa = 0.93), and clinician diagnoses of ADHD converged with diagnoses made via semi-structured diagnostic interviews (Kiddie Schedule for Affective Disorders and Schizophrenia-Epidemiologic Version; KSADS-E; [16–18]).

### Cognition

Major domains of cognition, measured by the Wechsler Intelligence Scale for Children—5th edition [27], were included as predictors of pandemic-related variability (see analytic plan below). Specifically, we examined General Ability, Processing Speed and Working Memory Indices, given the relevance of higher order cognition to adaptation to environmental demands and positive psychosocial adjustment [28].

### Analytic strategy

#### Comparison of pre-pandemic and current functioning on specific dimensions

First, we aimed to determine which individual domains of youth functioning showed statistically significant change between the time period prior to the pandemic and the time of evaluation. We used a Wilcoxon signed-rank test to examine parent retrospective ratings on the four-point severity scale (0 = not an issue, 1 = mild problem, 2 = moderate problem, 3 = severe problem) on 14 *a priori* selected dimensions. These included eight dimensions reflecting common aspects child psychopathology, one dimension related to substance use and five dimensions related to other aspects of psychosocial functioning.

#### Identification of patient groups with similar patterns of symptom change

Second, we conducted a Latent Profile Analysis (LPA) to classify children into groups (i.e. latent classes) based on their pattern of multivariate symptom change on the eight psychopathology domains. Change scores were based on changes in symptom severity scores noted above. In cases where the level of symptom severity was the same pre-pandemic and during the pandemic, the c-question for each item (reflecting worse, same, or better) was incorporated into the change score by adding (if better) or subtracting (if worse) 0.5 points to the difference score in order to increase sensitivity to change. This analysis, conducted using Mplus-version 8 [29], aimed to identify subgroups of individuals with similar patterns of change across symptoms, such that members within a class were more statistically similar to one another than to those in other classes.

#### Predictors and correlates of patient groups

Finally, we examined potential predictors and correlates of latent class membership (i.e. predictors of different patterns of change), including: a) prior psychopathology; b) other child-related characteristics and features of the family environment; and c) current correlates. Here we used multinomial logistic regression models to determine which, if any, retrospectively reported pre-pandemic factors or factors concurrent with the assessment predicted membership in classes that showed change compared to the class showing the least change. These models employed a Likelihood Ratio Test to statistically compare a base model including potential confounders (i.e. age, sex, date of assessment [months since the pandemic onset]) with a full model that included potential confounders plus predictor of interest. Based on this comparison, we extracted the delta pseudo R^2^ as an indicator of the effect size of the association between the predictor of interest and latent classes after controlling for potential confounders.

Potential predictors included the following, with variables dichotomized, as present/ absent prior to the pandemic:

*Psychopathology-related*: parent rated symptoms of the eight psychopathology domains (any, whether mild moderate or severe) and clinician diagnoses of ADHD or ASD.

*Other child-related* *and aspects of the child’s environment*: Child cognitive functioning (General Ability, Working Memory and Processing Speed); being on an educational (IEP or 504) plan prior to the pandemic; having a negative experience with remote learning in spring 2020; family worried about paying bills; child, close friend or family member diagnosed with COVID.

*Change variables since the start of the pandemic*: Increased job insecurity, increased screen time, increased arguing/conflict with parents, increased desire for interaction and feelings of isolation, decreased time exercising.

We used the Mplus program-version 8 [29] to conduct the latent profile analyses. Per convention, Akaike Information Criteria (AIC), Bayes Information Criteria (BIC), sample size adjusted BIC, entropy, Lo-Mendell-Rubin Adjusted Likelihood Ratio Test (LRT), and bootstrapped LRT were used to determine the number of distinct classes that best fit the data. For all other analyses, we used STATA version 14. These analyses controlled for age, sex and number of months since the onset of the pandemic (per the World Health Organization, March 11, 2020). Conservative Bonferroni corrected p-values were used in the analyses of individual symptom changes and predictors of group membership. Specifically, we used 0.0036 (0.05/14) analyses for the comparisons between prior and current severity of individual domains [2, 6, 22–24]. To determine the variables associating with LPA group membership, a corrected p-value of 0.005 (0.05/10 analyses) was used for the psychopathology related analyses, a corrected p-value of 0.007 (0.05/7 analyses) was used for other child-related characteristics and features of the family environment*,* a corrected p-value of 0.01 (0.05/5 analyses) was used for change variables.

## Results

In the 6-month period of our data collection, parents of 171 unrelated clinically referred youth, ages 6–17 filled out our survey. This group represented a response rate of 69% of parents who were approached to fill out the survey from our source study during that time frame. Table 1 illustrates the demographic and diagnostic characteristics of the sample. The mean age was 10.6 ± 3.1 years, 39.2% are girls and the average time of assessment from start of the pandemic (March 14th) was approximately 10.7 ± 1.8 months. Rates of major diagnostic categories and comorbidity in the sample (shown in Table 1) are generally comparable to the proportions found in our larger, source study from which these youth were surveyed. Details regarding the largest groups are as follows: The rate of ADHD was 67.3% (n = 115), including n = 77 Combined Type and n = 38 Inattentive Type. Within the mood disorders category, diagnoses included: 15.8% (n = 24) major depressive disorder/ other specified depressive disorder, 2.9% (n = 5) bipolar disorder/ other specified bipolar and related disorders), 1.2% (n = 2) persistent depressive disorder, and 2.9% (n = 5) disruptive mood dysregulation disorder. Within the anxiety domain, the sample included: 19.3% (n = 33) generalized anxiety disorder (GAD), 0.6% (n = 1) social anxiety disorder, 0.6% (n = 1) separation anxiety disorder, and 20.5% (n = 35) other specified anxiety disorder.

**Table 1:** Characteristics of sample (n = 171 youth, ages 6–17)

Variable	Descriptive
Age; M (SD)	10.6 (3.1)
Time of assessment (months since pandemic onset); M_months_ (SD)	10.7 (1.8)
Full Scale IQ; M (SD)*	96.2 (6.1)
Sex; N_girls_(%)	67 (39.2)
Race	N (%)
White	143 (83.6)
Black/ African American	9 (5.3)
Asian	5 (2.9)
Native Hawaiian/ Other Pacific Islander	1 (0.6)
Other/ Mixed	13 (7.6)
Ethnicity	
Hispanic/ Non-Hispanic	13/ 156 (7.6/ 91.2)
Missing	2 (1.2)
Lifetime diagnosis	N (%)
ADHD	115 (67.3)
ASD	25 (14.6)
Anxiety Disorders	70 (40.9)
Mood Disorders	36 (21.1)
Psychosis	6 (3.5)
Number of (neuro)psychiatric diagnoses	
0	21 (12.9)
1	79 (46.2)
2	43 (25.1)
3	21 (12.3)
4	6 (3.5)

* = based on n = 163

### Comparison of pre-pandemic and current functioning on specific dimensions

**Fig. 1:**
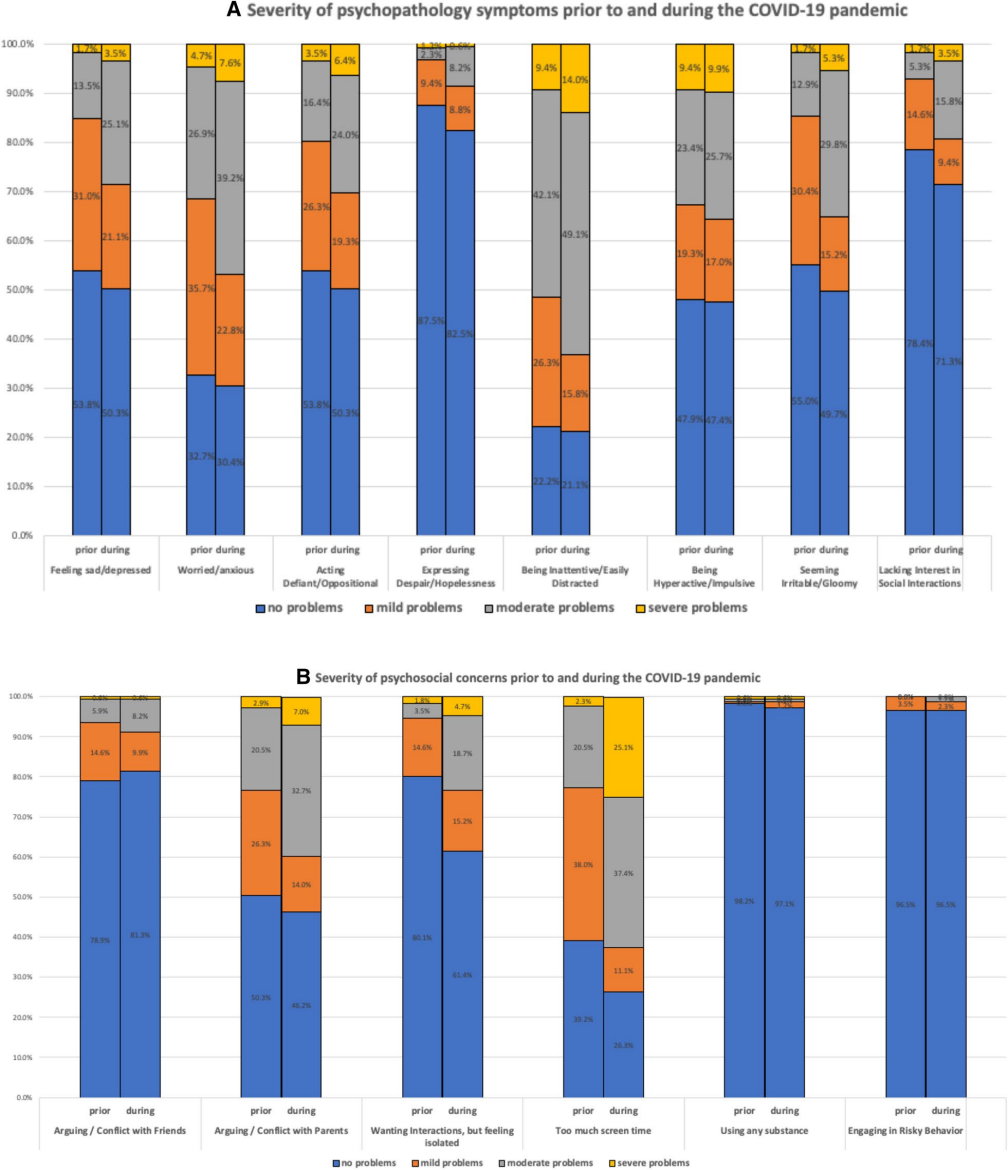
Comparison of pre-pandemic and current functioning on specific dimensions

Figure 1a illustrates the frequencies of symptom severity on the eight symptoms of psychopathology which were retrospectively reported prior to the pandemic as well as at the time of assessment. Wilcoxon signed-rank tests show significant worsening of all psychopathology symptom domains, with the exception of “hyperactive/impulsive.” Sixty-one percent of the parents reported a negative change on one or more of the eight psychopathology domains. More specifically, 26.9% reported negative change on 1 or 2 domains, 17.0% on 3 or 4 domains and 17.0% between 5 and 8 domains. In Fig. 1b, which illustrates the prior and current frequencies of the other six individual dimensions, significant worsening occurred on three domains, including “arguing/conflicts with parents,” “wanting interactions, but feeling isolated,” and “spending too much time on electronic devices”. While no changes were found on substance use and engaging in risky behavior, we note that the base rate for these latter behaviors in the sample was low (Fig. 1b).

### Identification of patient groups with similar patterns of symptom change

**Fig. 2:**
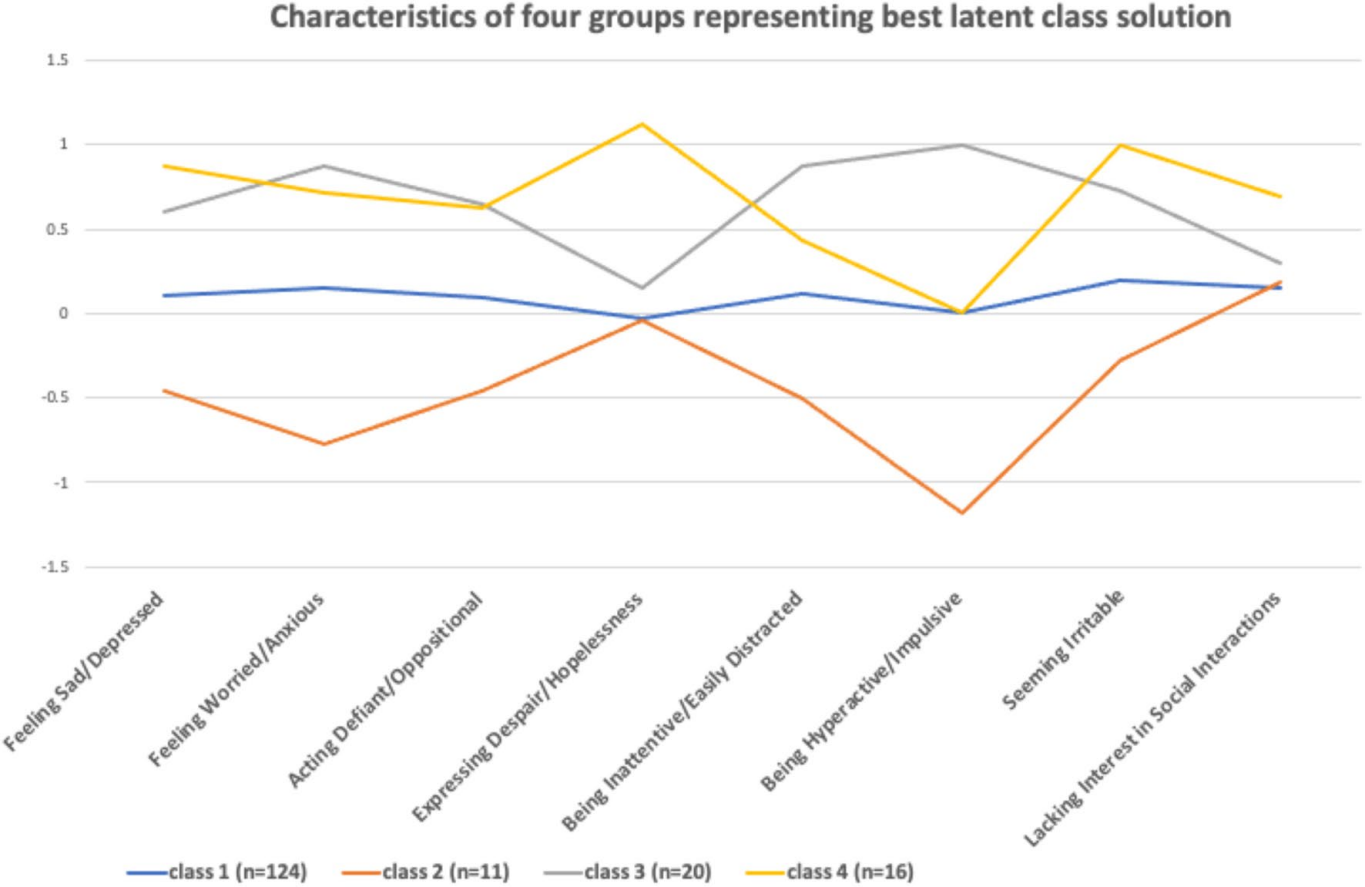
Characteristics of four groups representing best latent class solutions

In the latent profile analysis, the 4-class solution showed the best goodness-of-fit statistic (Table 2). The AIC and BIC for this solution was lower than in the 3-class solution. Entropy was close to 1, and, although the Lo Mendell Rubin Likelihood Ratio Test comparing the 4-class solution with the 3-class solution was not significant, the bootstrapped Likelihood ratio test comparing the 4-class with the 3-class solution was significant (see Table 3 for details). Figure 2 illustrates the features of the groups identified in the 4-class solution. The largest class (heretofore group 1), represented 72.5% (n = 124) of the children where youth showed relatively minimal change. The smallest class (group 2), represented 6.4% (n = 11) of youth and was most notably characterized by improvements on some symptom domains (predominantly on the sad/depressed, worried/anxious, and hyperactive/impulsive dimensions). Additionally, there were two distinct classes showing a worsening of symptoms. One of these (group 3), representing 11.7% (n = 20) of the sample, showed a pattern of worsening of most symptoms, particularly worse hyperactivity/impulsivity and, but with minimal change in “despair/hopelessness” and “lacking interest in social interactions”. The other group characterized by worsening symptoms (group 4), consisting of n = 16 children (9.4%), also showed a general worsening of a range of symptoms. Here, with the most salient features of change related to worse “expressing despair/hopelessness” and “seeming irritable” but minimal change on the domain of “hyperactive/impulsive” behavior.

**Table 2:** Goodness of fit statistics of the different latent profile analyses

Goodness of fit measures	1 class	2 classes	3 classes	4 classes	5 classes
AIC	2327.75	2110.31	1868.65	**1720.00**	Did not converge
BIC	2378.02	2188.85	1975.46	**1855.09**	
Sample adjusted BIC	2327.36	2109.69	1867.80	**1718.94**	
Entropy	–	0.86	1.000	**0.998**	
LMR	–	2 vs 1	3 vs 2	**4 vs 3**	
	–	230.46	254.17	**163.75**	
P –value	–	0.0005	0.72	**0.37**	
Bootstrapped LRT	–	235.45	259.66	**166.64**	
	–	< 0.0001	< 0.0001	** < 0.0001**	

*AIC* Akaike Information Criterion, *BIC* Bayesian Information Criterion, *LMR* Lo, Mendell, Rubin Likelihood Ratio Test. LRT = Likelihood Ratio Test. Bold data = solution that best fit the data

**Table 3:** Association between pre-pandemic psychopathology and latent class membership controlling for age, sex, and time since pandemic onset (n = 171)

	Better versus no change			Worse (hyperactive) group versus no change			Worse (despair) group versus no change			Δ pseudo R^2^(%)	LRT χ^2^(3)	p-value
	RRR	95% CI	P	RRR	95% CI	P	RRR	95% CI	P			
Early onset diagnoses												
ADHD dx	0.85	0.23–3.15	0.81	1.90	0.63–5.80	0.26	2.58	0.69–9.73	0.16	1.15	3.47	0.32
ASD dx	2.31	0.52–10.32	0.27	0.90	0.18–4.51	0.90	0.77	0.15–3.85	0.75	0.44	1.33	0.72
Prior symptoms (yes/no)												
Sad/Depressed	2.04	0.55–7.56	0.29	1.54	0.54–4.39	0.42	3.01	0.93–9.75	0.07	1.54	4.67	0.20
Worried/Anxious	3.26	0.65–16.24	0.15	2.46	0.74–8.15	0.14	2.50	0.66–9.44	0.18	1.96	5.91	0.12
**Defiant/Oppositional**	**1.72**	**0.49–6.01**	**0.40**	**6.40**	**1.97–20.82**	**0.002**	**1.61**	**0.56–4.63**	**0.38**	**4.06**	**12.27**	**0.007**
**Despair/Hopelessness**	**3.85**	**0.67–22.04**	**0.13**	**3.77**	**0.94–15.09**	**0.06**	**14.88**	**4.23–52.37**	**< 0.001**	**6.31**	**19.03**	**< 0.001**
Inattentive/Easily Distracted	1.52	0.31–7.52	0.61	3.27	0.70–15.30	0.13	2.55	0.54–12.01	0.24	1.41	4.25	0.24
** Hyperactive/Impulsive**	**All 11 had prior hyperactivity**			**11.16**	**2.84–43.86**	**< .001**	**2.99**	**0.96–9.32**	**0.06**	**8.42**	**18.52**^†^	**< .001**
Seeming Irritable	4.89	1.20–19.89	.027	2.35	0.87–6.37	0.09	2.64	0.89–7.82	0.08	3.25	9.80	0.020
Lacking Interest in Social Interactions	1.46	0.35–6.09	0.60	0.74	0.19–2.81	0.66	1.62	0.50–5.24	0.42	0.39	1.17	0.76

Bold data = solution that best fit the data

*LRT* Likelihood Ratio Test, *RRR* Relative Risk Ratio, *CI* Confidence Interval; ^†^df = 2

### Predictors and correlates of patient groups

Tables 3, 4, and 5 show the results of the multinomial logistic regression analyses, with relative risk ratios (RRR) reported with the “no change group” as reference category, along with the delta pseudo R^2^, and Likelihood Ratio test statistics.

**Table 4:** Association between other child-related characteristics and features of the family environment and latent class membership controlling for age, sex, and time since pandemic onset (n = 171)

	Better vs. no change			Worse (hyperactive) group vs. no change			Worse (despair) group vs. no change			Δ pseudo R^2^(%)	LRT χ^2^(3)	p-value
	RRR	95% CI	P	RRR	95% CI	P	RRR	95% CI	P			
Child-related features												
Cognitive functions												
GAI*	1.01	0.96–1.05	0.72	0.98	0.94–1.02	0.26	1.03	0.99–1.07	0.19	1.57	3.59	0.31
Working Memory**	0.95	0.91–1.00	0.039	0.98	0.95–1.02	0.32	1.03	0.99–1.06	0.15	3.03	8.32	0.04
Processing Speed***	0.98	0.94–1.03	0.44	1.02	0.98–1.05	0.32	1.02	0.98–1.06	0.36	0.91	2.52	0.47
Negative remote learning experience spring 2020	2.71	0.55–13.42	0.22	1.08	0.39–2.94	0.89	1.08	0.36–3.25	0.89	0.58	1.75	0.63
Child on IEP or 504 plan	1.48	0.39–5.53	0.56	2.79	1.02–7.63	0.046	2.37	0.79–7.08	0.12	1.86	5.62	0.13
Features of the child’s environment												
Family worried about paying bills	0.37	0.08–1.82	0.22	1.74	0.65–4.60	0.27	2.81	0.96–8.26	0.06	2.28	6.87	0.08
Close family friend diagnosed with COVID	0.76	0.18–3.16	0.70	0.73	0.25–2.18	0.58	0.99	0.29–3.38	0.99	0.14	0.42	0.94

*LRT* Likelihood Ratio Test, *RRR* Relative Risk Ratio, *CI* Confidence Interval; * n = 132, ** n = 161, *** n = 158

**Table 5:** Association between change variables on latent class membership controlling for age, sex, and pandemic duration (n = 171)

	Better versus no change			Worse (hyperactive) group versus no change			Worse (despair) group versus no change			Δ pseudo R^2^(%)	LRT χ^2^(3)	p-value
	RRR	95% CI	P	RRR	95% CI	P	RRR	95% CI	P			
Increased job insecurity	1.30	0.31–5.52	0.72	3.29	1.14–9.48	0.03	3.96	1.26–12.38	0.02	2.97	8.98	0.030
Increased screen time	0.62	0.17–2.25	0.46	4.78	1.46–15.69	0.01	4.57	1.24–16.90	0.02	4.77	14.41	.002
Increased conflict w/ parents	None of these 11 became worse			13.98	4.47–43.72	< 0.001	2.75	0.90–8.40	0.08	11.57	25.45^†^	< 0.001
Increased feelings of isolation	1.91	0.51–7.12	0.34	5.15	1.83–14.49	0.002	3.90	1.33–11.50	0.01	4.77	14.39	.002
Decreased exercise	0.74	0.21–2.58	0.63	1.67	0.61–4.57	0.32	2.80	0.85–9.27	0.09	1.43	4.32	0.23

*LRT* Likelihood Ratio Test, *RRR* Relative Risk Ratio, *CI* Confidence Interval; ^†^ df = 2

For the psychopathology dimensions shown in Table 3, the presence of two types of symptoms prior the start of the pandemic were significantly associated with class membership after Bonferroni correction. Prior evidence of hopelessness/despair increased the risk of being in group 4 (the group with worse despair/hopelessness) 14.9-fold compared to the class showing minimal change (group 1). Prior symptoms of hyperactivity/impulsivity significantly increased the risk of being in group 3 (the group with the worst hyperactivity) versus class 1 by 11.2 fold. We cannot, however, rule out a relationship between hyperactivity and other classes. For example, the relationship with that symptom fell just short of significance for Class 4, and all 11 children in group 2 (characterized by improving symptoms) had prior symptoms of hyperactivity/impulsivity. Additionally, prior symptoms of acting defiant/oppositional increased the risk of being in group 3 (with worsening hyperactivity) 6.4-fold compared to class 1 (the minimal change group); however, this finding did not survive Bonferroni correction. Diagnoses of ADHD and ASD were not predictive of class membership.

Table 4 shows the results of the multinomial logistic regression analyses examining the relationship between child-related characteristics and aspects of the child’s environment with group membership. No significant associations were found for cognitive functions, being on a special education plan, having a negative experience with remote learning, pre-existing worry about paying the bills or having the child or close family member/friend diagnosed with COVID.

Among the five change variables, increased screen time was significantly associated with increased risk of being in group 3 (4.8-fold) and group 4 (4.6-fold) compared to being in the no change group. Similarly, feelings of isolation increased the risk of being in group 3 by 5.2-fold and group 4 by 3.9-fold. An increase in arguing/conflict with parents increased the risk for being in the hyperactive group (group 3) by 14.0-fold and showed a non-significant trend towards association with group 4. Spending less time on exercise was not significantly related to group membership.

## Discussion

Studies from across the globe are documenting the impact of the COVID-19 pandemic on youth mental health. We extend this literature in several ways. Specifically, we document increased severity of a wide range of psychiatric symptoms within a generalizable child psychiatry outpatient sample in the United States and at a time period well beyond the 2020 spring lockdown. Further, we idenitfied distinctive profiles of psychiatric symptom change among referred youth. Encouragingly, between the pre-pandemic period and the mid 2020–2021 school year, a large group showed minimal difference in symptom severity. Additionally, a small group was characterized by improved symptoms; however, two groups had differing profiles of worsening symptoms, with shared and unique predictors and correlates. Such data confirm that the impact of the ongoing pandemic on youth is not uniform, even within a clinical sample and even among those who are struggling. As such, supports will need to be tailored to the unique needs of those who remain in distress.

Our first set of analyses focused on changes in individual psychiatric and psychosocial domains. Based on parent reports, over two thirds of the sample worsened on at least one of eight psychopathology domains. On average in the overall sample, seven domains showed a significant increase in severity, including: sad/depressed, worried/anxious, despair/ hopelessness, inattentive/ easily distracted, irritable/ gloomy, and lacking interest in social interactions. Consistent with adolescent self-reports in a UK survey [30], which spanned the time prior to the pandemic and July 2020, hyperactive/impulsive symptoms failed to show a significant change on average within the whole sample, though other studies [31] found increases in this domain when measured at an earlier time point in the pandemic.

Our US-based clinical sample is consistent with clinical cohorts from Canada [10] and the Netherlands [32] in showing change on a wide range of domains. Additionally, while other studies of clinical cohorts documented such symptoms in spring 2020, our results suggest that this increased symptom severity was present at even a later time, during the middle of the 2020–2021 school year. In the US, while the most extreme period of school closures and social isolation had abated by that point, significant psychosocial and educational disruption was still occurring [33]. In contrast to evidence for a bounce-back effect for adults in China a month after the pandemic onset [34], our results showed that psychiatric symptoms in clinically referred youth in the US were worse during a time period that was 10 months on average after the pandemic onset. Our finding that youth were impacted at this later date converges with longitudinal data from a population youth cohort in Germany [35] showing that increases in individual symptom domains were observed in winter of the school year following the pandemic. While youth from a population cohort in Denmark [36] did not identify significant depression symptoms in the overall group in fall of that year, they did note that youth with high pandemic related anxiety in the prior spring experienced greater depressive symptoms in the fall. This result is consistent with our findings of heterogeneity and of a relationship between functioning during the ongoing pandemic and prior symptoms.

Parent retrospective reports also indicated greater conflict between parents and children and, unsurprisingly, increased feelings of isolation in youth. Parents further reported a higher level of excessive screen time compared to the pre-pandemic period. Although our data are not longitudinal, the change from baseline into the 2020–2021 school year suggests the potential for prolonged exposure and that potential vulnerability to internet addiction and other negative effects of screen time should be followed up [37]. We did not find worsening conflict with friends or increased use of alcohol or subtances during this time, likely due to the limited social opportunities outside of the home.

When we used LPA to characterize the heterogeneity of responses within our sample, participants segregated into four groups with statistically distinct profiles of symptom change. As noted, the majority of youth showed minimal change. We do not know whether members of this group had previously experienced greater distress at the height of the lockdown in spring 2020. Indeed, based on parents free-form verbal impressions of the impact of the pandemic, Asbury et al. [38] posited that only a small subgroup of youth with ADHD and neurodevelopmental disorders had been unchanged/improved at that time. Nonetheless, it is encouraging that, even within a clinical sample, the majority of youth were relatively un-changed in their level of psychiatric symptoms during the following school year. This is not to say that the pandemic was not still challenging for these children nor that they didn't have psychiatric symptoms. Rather, our data speak to the fact that substantial increases in psychiatric symptoms during the 2020–2021 school year were not universal among referred youth.

Yet, the LPA also identified three subgroups whose psychiatric symptom profiles had changed compared to the pre-pandemic period. A small group of youth (6%) showed improved functioning. There were also two distinct groups with worsening symptoms. Both experienced increases in sadness/depression and worry/anxiety, consistent with studies from spring 2020 suggesting that these are some of the most common sequelae of the pandemic in youth [20, 39]. Both groups also increased in their defiance/ oppositionality. Yet, members of one group exhibitied significant worsening of symptoms most commonly related to ADHD, particularly hyperactivity/ impulsivity but also inattention, and these youth showed minimal change in hopelessness/despair. In contrast, the other worse group showed notably increased symptoms linked to depression, including hopelessness/despair and social withdrawal with minimal change in hyperactivity/ impulsivity.

Although two prior studies [40, 41] identified heterogeneous groups (based on profiles of behaviors and pandemic-related life changes, respectively) in non-clinical samples, our identification of distinct patterns of psychiatric symptom change within a clinical sample of youth is novel and helps to integrate discrepancies across prior studies showing worsening [8] and improving symptoms or the worsening and improving of individual traits. By using overall child functioning rather than individual traits as our outcome, we found that different children were exhibiting globally distinctive patterns of stability, improvement and worsening by the mid 2020–2021 school year.

We also found that specific types of problematic symptoms prior to the pandemic associated with change profiles relevant to those symptoms. Prior hopelessness was associated with membership in the worsening group that stood out for its depressive symptoms, while prior conduct problems predicted the group with worsening hyperactivity/impulsivity with no increase in hopelessness. Such findings echo prior studies [13, 41, 42] that suggest a worsening of prior symptoms.

We examined variables that had not been examined in prior pandemic related studies, including (1) clinician-rated lifetime diagnoses of ADHD and autism (rather than parental reported diagnoses) and (2) cognitive measures reflecting general ability, working memory, and processing speed. None of these variables were associated with change profiles. The lack of association with cognition was somewhat surprising given that general ability and executive functions are known to relate to successful problem solving and psychosocial adjustment [43]. However, results are consistent with growing evidence for the separability of cognitive functioning and psychopathology per se [44] and do not speak to the important question of whether cognition may relate to pandemic related adjustment within academic domains specifically. Additionally, having previously been on a special educational plan (504 or IEP) did not significantly associate with group membership; however, it is notable that the group whose functioning improved, presumably because they were experiencing less academic and social stress during the pandemic, showed the largest effect size for this variable.

We also found that certain change variables related to the groups with worsening symptoms. Increased screen time and increased isolation were significantly associated with increased risk of being in each of the two worse groups compared to the no change group. Increases in arguing/conflict with parents increased the risk for being in the hyperactive group. Because these variables changed over the same time period as the symptoms did, we cannot presume causality and must consider them correlates. Nonetheless, they do underscore the need for studies to determine whether reducing social isolation, excessive screen time and conflict could provide relief to youth who experienced worsening symptoms.

The current results represent an important step towards understanding the impact of the pandemic on clinical samples. Certainly, the toll on youth mental health generally is clear, and the impact of the ongoing disruption of social and educational structures has yet to be fully characterized. Nonetheless, our data provide arguably the clearest empirical evidence to date of the variability within a child and adolescent clinical sample, including evidence that subsamples of youth experienced significantly increased and distinguishable psychiatric symptoms that parents attributed to the pandemic as recently as the 2020–2021 school year. Given provider shortages in child psychiatry that existed even prior to the pandemic [28] compounded with the continued burden of educational disruption, identifying the unique needs of youth at highest risk may allow for more targeted and effective care. This is not to say that programs targeting general wellness are not of value. Indeed, Copeland et al. [45] found a small but significant benefit to the mental health of college students enrolled in a neuroscience based wellness program during the pandemic. Nonetheless, in clinical samples, youth who experienced notably increased hopelessness during the last school year in addition to other symptoms may require a different response than youth whose symptoms improved during the period of reduced interactions in academic and social settings.

Our findings should be considered in light of their limitations. Our assessment of the pre-pandemic period was based on parental retrospective reports and prospective data would have improved the accuracy of reports about the pre-pandemic period. Additionally, youth were assessed at different times during a six month period and, even though we controlled for the time of assessment, we cannot rule out the possibility that these differences contributed some variability to our sample. Importantly, the groups showing changes in symptoms were small, and the effect sizes for some variables (e.g. prior 504/IEP plan, irritability, increasing job insecurity) suggest that there may be additional predictors and correlates of group membership that we were not able to identify due to Type II error. Additionally, we acknowledge that the time frame of our assessment does not speak to functioning during the current 2021–2022 school year, and futher data on the current time period are needed. Finally, the fact that our sample is predominantly White creates uncertainty with regard to the generalizability of our findings to youth from other racial and ethnic groups who may have experienced additional or different stressors during the pandemic. For example, there is growing evidence for structural inequities that may place burdens on BiPOC (Black, Indigenous, and People of Color) youth that were compounded by the pandemic [46]. Examination of clinical cohorts with greater racial and ethnic representation is needed to determine the relevance of our results to referred youth from these historically under-represented groups.

## Conclusion

Despite the above limitations, our data provide evidence for the variability of the mental health impact of the pandemic within an outpatient child psychiatry sample in the United States during the middle of the 2020–2021 school year. Within that year following the pandemic onset, a wide range of psychiatric and psychosocial difficulties had worsened on average in our clinical cohort, consistent with prior studies; yet, distinctive patterns of symptom change were also identified. While a large group of youth showed minimal difference in symptom severity and a small group showed improved symptoms, two groups showed worsening of a range of symptoms. Both had greater depressive and anxiety symptoms, but one had worsening hopelessness and the other had worsening hyperactive/inattentive symptoms. Group membership was predicted by prior symptoms from relevant psychopathology domains and correlated with increases in screen time, conflict with parents, and feelings of isolation. These findings suggest that different groups of outpatients may have unique needs as they emerge from this period. Results highlight the importance of considering the global functioning of the child by assessing multiple psychiatric domains simultaneously in order to identify youth in greatest distress and their distinctive patterns of symptoms that may be targets for clinical care.

## Supplementary Information

**Additional file 1: Appendix S1.** COVID-19 Survey Questions.

## Data Availability

The dataset generated and analyzed during the current study is not publicly available currently (due to constraints based on participant consent), but will be placed in an NIH repository upon the completion of the R01 that is funding current data collection.

## Abbreviations

ADHD: Attention Deficit Hyperactivity Disorder
AIC: Akaike Information Criteria
ASD: Autism Spectrum Disorder
BIC: Bayes Information Criteria
BiPOC: Black, Indigenous, and People of Color
DSM-5: Diagnostic and Statistical Manual of Mental Disorders, 5th Edition
IEP: Individual Education Plan
LOGIC: The Longitudinal Study of Genetic Influences on Cognition
LPA: Latent profile analysis
LRT: Lo-Mendell-Rubin Adjusted Likelihood Ratio Test
MGH: Massachusetts General Hospital
REDCap: Research Electronic Data Capture
