# Supplementary material for: Distinct patterns of emotional and behavioral change in child psychiatry outpatients during the COVID-19 pandemic
Source: Child Adolesc Psychiatry Ment Health. 2022 Feb 17;16:12. doi: 10.1186/s13034-022-00441-6 (PMC8851810; doi:10.1186/s13034-022-00441-6)
Supplement: Supplementary file 1 — Additional file 1: Appendix S1. COVID-19 Survey Questions. [file 13034_2022_441_MOESM1_ESM.docx]

| **Appendix 1. COVID-19 Survey Questions**  **Psychiatric and Psychosocial Domains** | | |
| --- | --- | --- |
| **Has your child ever had issues with the following:** | | |
| 1 | Feeling Sad / Depressed / Down | 1, Yes \| 2, No |
| 2 | Feeling worried/ anxious | 1, Yes \| 2, No |
| 3 | Acting defiant/ oppositional | 1, Yes \| 2, No |
| 4 | Expressing despair / hopelessness | 1, Yes \| 2, No |
| 5 | Being inattentive/ easily distracted | 1, Yes \| 2, No |
| 6 | Being hyperactive/ impulsive | 1, Yes \| 2, No |
| 7 | Seeming irritable/grumpy | 1, Yes \| 2, No |
| 8 | Arguing / conflict with friends | 1, Yes \| 2, No |
| 9 | Arguing / conflict with parents | 1, Yes \| 2, No |
| 10 | Lacking interest in social interactions | 1, Yes \| 2, No |
| 11 | Wanting interactions but feeling isolated | 1, Yes \| 2, No |
| 12 | Spending too much time on electronic devices | 1, Yes \| 2, No |
| 13 | Using marijuana | 1, Yes \| 2, No |
| 14 | Using alcohol | 1, Yes \| 2, No |
| 15 | Vaping/ using nicotine products | 1, Yes \| 2, No |
| 16 | Engaging in risky behavior | 1, Yes \| 2, No |
| **[If YES for each item 1-16]** | | |
| [#]a | During the school year prior to the pandemic (i.e. September 2019 - February 2020) these issues were: | 1, Not an issue prior to the pandemic \| 2, Mild \| 3, Moderate \| 4, Severe |
| [#]b | Currently, these issues are: | 1, Not an issue currently \| 2, Mild \| 3, Moderate \| 4, Severe |
| [#]c | How have these changed since the onset of the pandemic (from March 2020 to current)? | 1, Gotten worse \| 2, Stayed the same \| 3, Gotten better |
| **Proximity to COVID 19 Diagnoses** | | |
| 20 | Has a close family member or friend been diagnosed with COVID- 19? | 1, Yes \| 2, No |
| 20a | Was your child diagnosed with COVID-19?* | 1, Yes \| 2, No |
| 20b | *[If YES to 20a.]* When were they diagnosed? | 1, Less than 1 month ago \| 2, 1-2 months ago \| 3, 3-4 months ago \| 4, 5 or more |
| **Job and Financial Security** | | |
| **21 To what extent were you or your spouse/live-in partner worried about the following during the school year prior to the pandemic (i.e. September 2019 - February 2020)?** | | |
| 21a | Job security | 1, Not concerned \| 2, Mildly concerned \| 3, Concerned \| 4, Extremely concerned |
| 21b | Paying bills | 1, Not concerned \| 2, Mildly concerned \| 3, Concerned \| 4, Extremely concerned |
| **22 To what extent are you or your spouse/ live in partner currently concerned about the following?** | | |
| 22a | Job security | 1, Not concerned \| 2, Mildly concerned \| 3, Concerned \| 4, Extremely concerned |
| **28 How much time per day does your child spend doing exercise (walking, running, playing sports, etc.)?** | | |
| 28c | How has this changed since the pandemic began? | 1, Spends less time now \| 2, Spends the same amount \| 3, Spends a little more time \| 4, Spends a lot more time |
| **Learning/ Academic** | | |
| 32 | How did your child feel about adjusting to remote learning in the spring? | 1, Very negative experience; had a very difficult time \| 2, Mildly negative experience; did not like it \| 3, Felt neutral \| 4, Felt positive about on-line learning; liked it compared to in-person school \| 5, Not applicable; at school full time |
| 34 | Did your child receive special education services or accommodations (IEP or 504 plan) in the spring of 2020? | 1, Yes \| 2, No |
| *This question was added to the survey after it was underway (n=77). | | |
